# The Determination of Polycyclic Aromatic Hydrocarbons (PAHs) with HPLC-DAD-FLD and GC-MS Techniques in the Dissolved and Particulate Phase of Road-Tunnel Wash Water: A Case Study for Cross-Array Comparisons and Applications

**DOI:** 10.3390/toxics10070399

**Published:** 2022-07-19

**Authors:** Hanne Vistnes, Nadine A. Sossalla, Anna Røsvik, Susana V. Gonzalez, Junjie Zhang, Thomas Meyn, Alexandros G. Asimakopoulos

**Affiliations:** 1 Department of Civil and Environmental Engineering, Norwegian University of Science and Technology (NTNU), S.P. Andersens veg 5, 7031 Trondheim, Norway; nadine.sossalla@ntnu.no (N.A.S.); thomas.meyn@ntnu.no (T.M.); 2 Department of Chemistry, Norwegian University of Science and Technology (NTNU), Høgskoleringen 5, 7491 Trondheim, Norway; annarosvik@hotmail.com (A.R.); susana.v.gonzalez@ntnu.no (S.V.G.); junjie.zhang@ntnu.no (J.Z.); alexandros.asimakopoulos@ntnu.no (A.G.A.)

**Keywords:** PAHs, GC-MS, HPLC-DAD-FLD, road-tunnel wash water, solid phase extraction, accelerated solvent extraction, forensic ratios

## Abstract

Accelerated solvent extraction (ASE) and solid phase extraction (SPE) protocols tailored to either gas chromatography mass spectrometry (GC-MS) or high-performance liquid chromatography coupled to diode-array and fluorescence detection (HPLC-DAD-FLD) were developed for the determination of EPA 16 polycyclic aromatic hydrocarbons (PAHs) in the particulate and dissolved phase of road-tunnel wash water. An analytical approach was developed, assessed, and applied on environmental samples collected from five road tunnels in Norway. The absolute recoveries ranged from 57 to 104% for the particulates, and from 42 to 79% for the dissolved water phase. The target PAH compounds were separated in 34.75 min using the GC method and in 22.50 min by HPLC. In the particulate phases, higher molecular weight PAHs were detected in the range of 0.043 to 0.93 µg/g, and lower molecular weight PAHs were detected in the range of 0.020 to 1.0 µg/g, while the intermediate ones were present in the range of 0.075 to 2.0 µg/g. In contrast to the particulates, the dissolved phase mainly contained lower molecular weight PAHs in the range of 0.0098 to 0.50 µg/L. GC-MS demonstrated lower detection limits (LODs) than HPLC-DAD-FLD for 13 out of the 16 PAHs. A cross-array comparison of the two analytical techniques indicated that some target PAHs were detected solely or in higher concentrations with HPLC-DAD-FLD, indicating the occurrence of false positive peaks or/and co-eluting components. The resulting concentrations in the road tunnel wash water samples were used to calculate specific PAH forensic ratios to pinpoint the potential sources of PAH pollution. These ratios revealed that there are several potential sources for the origin of PAHs in tunnel wash water.

## 1. Introduction

The pressure of chemical pollution is by default higher nearby urbanized areas. Organic and inorganic contaminants are released from a variety of sources, such as industrial facilities, wastewater treatment plants, and road traffic. Traffic emissions occur through different pathways, e.g., gas emissions from fuel combustion and particle generation from tire wear and tear [1,2]. Traffic emissions in road tunnels is a field of study that has gained high interest in recent decades. Although the presence and composition of contaminants in street runoffs is thoroughly studied, there is little information about contaminant loads in road tunnels. They are assumed to be higher in road tunnels due to their uniqueness in construction. In contrast to roads, which are exposed to meteorological phenomena such as rain or wind, the released contaminants in the tunnels accumulated on the roads, the inner walls, traffic signs, and the ceiling [3]. Therefore, for road safety and maintenance purposes, tunnels are regularly washed. The washing strategy depends on several factors, such as the location of the tunnel, the seasonal variation of the weather at the specific location, and the amount of traffic present [4]. The resulting tunnel wash water (TWW) typically contains metals, polycyclic aromatic hydrocarbons (PAHs), and other organic contaminants. In most cases, the resultant TWW is discharged into the environment, either directly or prior to treatment through municipal sewage systems.

PAHs are a class of persistent organic contaminants composed of two or more connected benzene rings. They are formed through the incomplete combustion of organic material [5,6] and are carcinogens [7]. Due to their structure, PAHs demonstrate low solubility in water; nonetheless, PAHs are also detected in aqueous matrices [5]. Water-dissolved PAHs are more bioavailable for organisms than those attached to particles [6]. This highlights the necessity to assess the PAH-dissolved phase of a water sample without undermining the contribution of the particulates. This can be achieved by calculating the total concentration of PAHs in a sample by summing up the PAH’s concentrations determined in the particulate phase of the water sample with those determined in the respective dissolved phase of the sample. Therefore, a suitable extraction method for PAH determination is required for both the particulate and dissolved phases. To extract PAHs from the particulate phase, an accelerated solvent extraction (ASE) is commonly applied, since it can combine the steps of extraction and clean-up of the sample preparation in a single process [8]. For the extraction of PAHs from the dissolved phase, solid-phase extraction (SPE) is mainly used for achieving extraction and clean-up in addition to preconcentration (particularly important for larger volumes of dissolved phase) [5]. Gas chromatography (GC) coupled to flame ionization, electron capture, or mass spectrometry (MS) detectors and liquid chromatography (LC) coupled to ultraviolet absorption diode-array detector (DAD) or fluorescence detectors (FLD) are commonly applied chromatographic techniques for PAH determination [5,9].

The concentration ratios between various PAH analogues in a sample can indicate the source of these contaminants, which are also known as PAH forensic ratios [10]. These ratios were established based on the varying occurrence of PAH analogues in the samples due to different emission sources. For example, low molecular weight (LMW) PAHs usually emerge from petrogenic sources, such as coal, crude oil, and refined oil products, while high molecular weight (HMW) PAHs are produced during the incomplete combustion of organic matter [11,12]. Co-assessing different types of such ratios can provide indications of the origin(s) of the PAH pollution pressures.

With this background, the present study aims to assess and compare the performance of two techniques, GC coupled with MS (GC-MS) and HPLC coupled with DAD and FLD (HPLC-DAD-FLD), for the analysis of 16 PAHs in the particulate and dissolved phases of road tunnel wash water. Moreover, an extraction protocol was developed and applied on wash water samples collected from five road tunnels in Norway. The objectives were the following: (1) compare the applicability of the two analytical procedures, (2) assess if the extraction procedures were suitable for the actual samples (fit-for-purpose), and (3) apply the forensic ratios to assess the potential source(s) of PAH contamination in a specific road-tunnel system.

## 2. Materials and Methods

### 2.1. Chemicals and Materials

Analytical standards (neat) of the 16 EPA PAHs (naphthalene (NAP), acenaphthylene (ACY), acenaphthene (ACE), fluorene (FLU), phenanthrene (PHE), anthracene (ANT), fluoranthene (FLT), pyrene (PYR), benzo(a)anthracene (BaA), chrysene (CHR), benzo(b)fluoranthene (BbF), benzo(k)fluoranthene (BkF), benzo(a)pyrene (BaP), dibenzo(a,h)anthracene (DBA), benzo(g,h,i)perylene (BgP), and indenol(1,2,3-cd)pyrene (IND) were purchased from (Sigma-Aldrich; Steinheim, Germany). A mix of fluorinated PAHs (1-fluoronaphthalene (F-NAP, 200 µg/mL), 4-fluorobiphenyl (200 µg/mL), 3-fluorophenanthrene (F-PHE, 200 µg/mL), 1-fluoropyrene (F-PYR, 200 µg/mL), 3-fluorochrysene (F-CHR, 200 µg/mL), and 9-fluorobenzo(k)fluoranthene (F-BkF, 100 µg/mL)) dissolved in toluene was obtained from Chiron (Trondheim, Norway). Individual standard stock solutions (10 mg dissolved in 10 mL solvent) of NAP, ACY, ACE, FLU, PHE, FLT, PYR, BbF, and DBA were prepared in acetonitrile, and BaP, CHR, BaA, BkF, IND, ANT, and BgP were prepared in toluene. A 10 mg/L standard mixture containing all target analytes (TA) was prepared by consecutive acetonitrile dilutions. Calibration standards of different concentrations (0.01, 0.1, 1, 2, 5 10, 20, 50, 75, 100, 200, 400, 1000, and 1500 µg/L) were prepared in both acetonitrile (for HPLC analysis) and ethyl acetate (EtAc; for GC analysis). The mixture of fluorinated PAHs (F-PAHs) was used as internal standard mixture (IS) and it was fortified relative to the samples prior to the extraction procedure. Toluene, dichloromethane (DCM), methanol, water, acetone, hydrochloric acid (HCl, 37%), acetonitrile, and ethyl acetate of HPLC grade were purchased from VWR Chemicals (Oslo, Norway). Glass microfiber filters (Whatman, 0.7 µm pore size, 47 mm diameter; Sigma–Aldrich; Steinheim, Germany) were purchased and combusted at 450 °C for 4 h to generate a pore size filter of <0.45 µm [13] for obtaining the particulates from the water samples. Polypropylene tubes (50 mL) were purchased from VWR (Oslo, Norway). Bondesil C18 powder was used as solid phase in the SPE cartridge, and was purchased from Agilent Technologies (Santa Clara, CA, USA). Empty pre-fritted SPE tubes (12 mL, 20 µm), polyethylene frits (20 µm), copper powder, activated alumina, and diatomaceous earth were acquired from Merck (Darmstadt, Germany). Cellulose filters for ASE (27 mm diameter, Type D28; Merck, Darmstadt, Germany) were purchased from Thermo Fisher Scientific (Waltham, MA, USA). Freeze drying was performed with a Christ freeze-dryer (ALPHA 1-2 LDplus; Osterode am Harz, Germany), while for the extraction of particulates, a Dionex ASE-150 Solvent Extractor (Thermo Scientific) was used.

### 2.2. Sampling Locations and Sample Treatment

**Figure 1:**
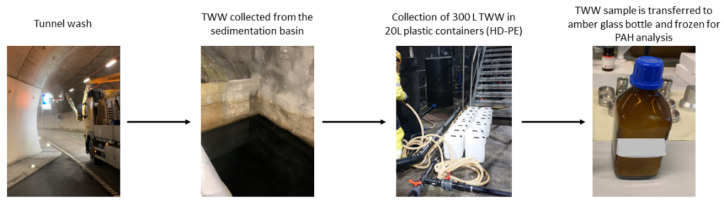
Schematic for sampling of tunnel wash water.

TWW samples were collected from five different road tunnels in Norway after their washing procedures between September 2020 and April 2021. A schematic of the sampling procedure is presented in Figure 1. More information of the sampling sites is presented in Supplementary Information, Table S1. The samples were collected in amber glass bottles and were kept frozen (−20 °C) until further analysis. For fraction separation, 50 mL of thawed TWW samples was filtered through glass microfiber filters to separate the dissolved phase from the particulate phase. The dissolved phase samples were acidified with 37% *v*/*v* hydrochloric acid (HCl) to pH ≤ 2 and stored at +4 °C until further analysis. Filters with the particulate phase were obtained through Büchner filtration, freeze dried for 24 h, and stored at room temperature.

### 2.3. Sample Preparation: Particulate Phase

**Figure 2:**
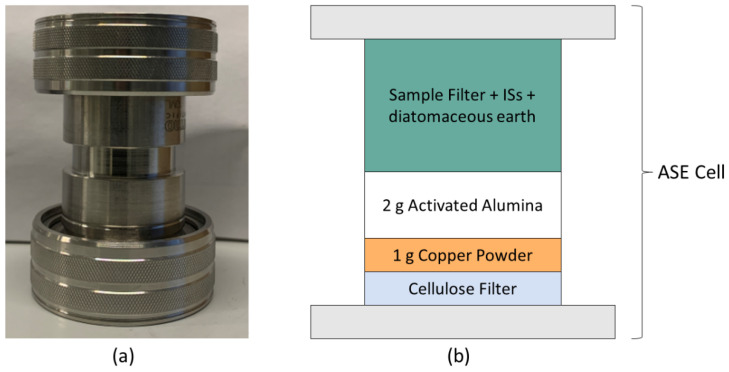
Description of the ASE extraction cell. (**a**) Extraction cell used for the ASE procedure. (**b**) Illustration of how the different layers of clean-up powders and sample are placed in the ASE extraction cell.

The extraction of PAHs from the particulate phase was performed with ASE as described by Sporring et al. [14] with minor modifications. Briefly, ASE cells were prepared by placing a cellulose filter in the bottom, followed by 1 g of copper powder and 2 g of activated alumina (Figure 2) to remove sulphur and nonpolar lipids from the sample matrix [15]. The particulate phase filter was cut into smaller pieces (maximum 7 × 2 mm), mixed with diatomaceous earth and fortified with IS. The sample was left under a fume hood until the solvent from the IS fortification was completely evaporated. Thereafter, the filter pieces were transferred to the ASE cell. PAHs were extracted by a solvent mix of dichloromethane and acetone (1:1 *v/v*) at a pressure of 1500 psi and a temperature of 100 °C. The complete set of parameters for the extraction is listed in Supplementary Information, Table S2.

**Figure 3:**
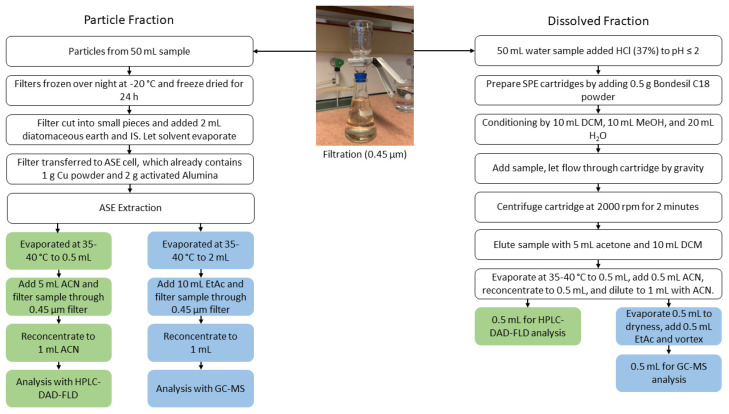
Schematic of the extraction procedure for particulate-bound PAH target analytes (**left side**) and the dissolved PAH target analytes (**right side**). The method for extraction of the dissolved phase PAHs was adapted from the EPA Method 8310 [16,17].

The extracts for HPLC-DAD-FLD and GC-MS analysis were evaporated at 40 °C to 0.5 and 2 mL, respectively. The solvent was added (5 mL acetonitrile for further HPLC-DAD-FLD analysis; 10 mL ethyl acetate for further GC-MS analysis), filtered with the 0.45 µm filters, and reconcentrated to 1 mL for HPLC-DAD-FLD and GC-MS analysis (Figure 3). The extracts were stored at−20 °C until analysis.

### 2.4. Sample Preparation: Dissolved Phase

The method was adapted from EPA Method 8310 [16,17]. A schematic of the procedure is presented in Figure 3. SPE cartridges (12 mL empty pre-fritted, 20 µm) were prepared for the extraction by adding 0.5 g Bondesil C18 powder in between two polyethylene frits into pre-fritted tubes. The cartridges were conditioned using 10 mL of DCM, followed by 10 mL of methanol, and equilibrated with 20 mL of HPLC grade water. Dissolved phase samples (50 mL) were added to the cartridges and a gravity-driven flow through the SPE was performed. The SPE cartridges were centrifuged to dryness (2000 rpm, 2 min) before eluting the respective extracts with 5 mL acetone followed by 10 mL DCM. The extracts were evaporated at 40 °C to 0.5 mL, 0.5 mL acetonitrile was added and reconcentrated to 0.5 mL, and diluted to a final volume of 1 mL with acetonitrile. The extract was split equally. One part was used for HPLC-DAD-FLD, and the other part was evaporated to dryness, reconstituted with 0.5 mL of ethyl acetate, and analyzed with GC-MS. All extracts were stored at −20 °C until analysis.

### 2.5. GC-MS Determination

**Figure 4:**
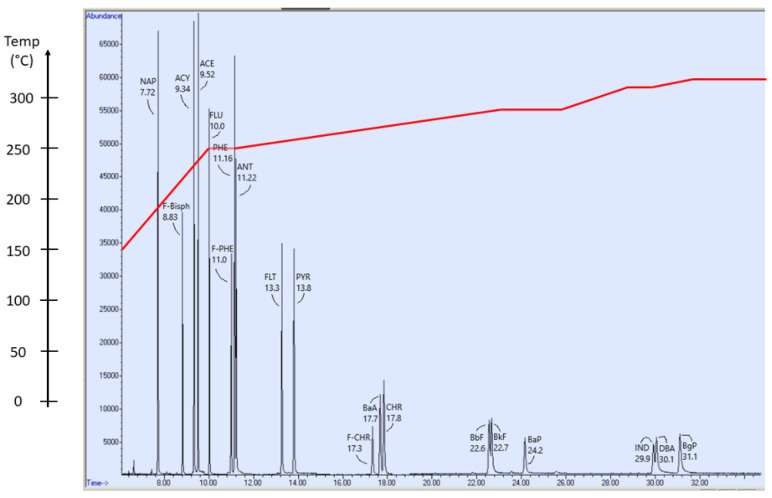
GC-MS chromatogram presenting the elution order of the 16 PAH target analytes. The sample was a calibration standard sample with a concentration of 100 µg/L of each PAH target analyte. The elution temperature gradient is presented superimposed on the chromatogram (red line). The oven gradient temperatures are listed in the Supplementary Information (Table S3).

The analysis of samples was performed with an Agilent 7890A gas chromatograph with a GC Pal autosampler (CTC Analytics, Zwingen, CH) coupled to an Agilent 5975 single quadrupole mass spectrometer. The separation of target compounds was performed on a Thermo Scientific™ TraceGOLD™ TG-5MS GC Column (5% diphenyl/95% dimethyl polysiloxane, 30 m × 0.25 mm inner diameter × 0.5 µm film thickness). The injection volume was set to 1 μL. In the column, helium served as the carrier gas with a flow of 1 mL/min. An oven temperature gradient was applied to separate the 16 PAH target analytes (Figure 4 and Supplementary Information, Table S3). Ionization was set to scan for specific m/z ratios in given time intervals (Supplementary Information, Table S4). The applied electron energy was set at 70 eV.

### 2.6. HPLC-DAD-FLD Determination

**Figure 5:**
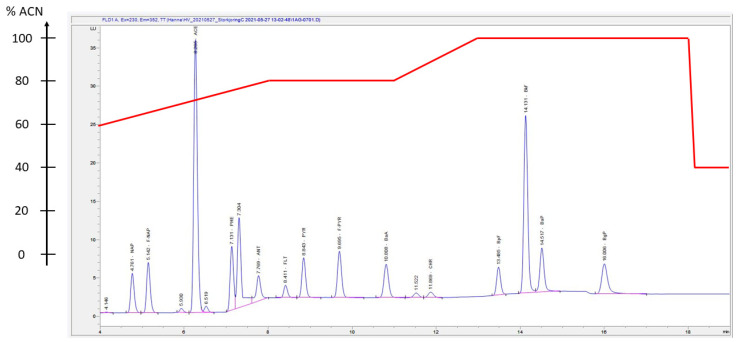
HPLC-FLD chromatogram presenting the elution order of 15 PAH target analytes. The sample was a calibration standard sample with a concentration of 100 µg/L of each PAH target analyte. Elution order: NAP, F-NAP, ACE, PHE, ANT, FLT, PYR, F-PYR, BaA, CHR, BpF, BkF, BaP, BgP. The elution solvent gradient, shown as percentage of acetonitrile (ACN) is presented superimposed on the chromatogram (red line). The solvent gradient percentages are listed in Supplementary Information (Table S5).

For HPLC-DAD-FLD analysis, an Agilent HPLC 1260 Infinity II LC System (Agilent, USA) was used. The system included an automatic sampler, a multicolumn thermostat, and detection was carried out by both a DAD and a FLD connected online. Separation was carried out by a Zorbax Eclipse PAH column (100 × 4.6 mm diameter, 1.8 μm). The HPLC-DAD-FLD analysis procedure was adapted from and inspired by several other procedures [18,19,20,21,22,23,24,25]. The injection volume was set at 5 μL, and a solvent gradient was used for the separation of the 16 PAHs (Figure 5 and Supplementary Information, Table S5, and Figure S1). The column temperature was kept constant at 20 °C throughout the analysis. The flow rate was set at 1.8 mL/min.

ACY is not detectable with FLD as it demonstrates weak fluorescence [26]; therefore, DAD was used for ACY quantification [27]. The DAD wavelength was set at 230 nm. The remaining 15 PAH target analytes were detected by the FLD detector, using the conditions presented in Table 1.

### 2.7. Extraction Performance

The extraction performance was assessed by conducting HPLC-DAD-FLD determination. Absolute recoveries were calculated for both the particulate and dissolved phases. This was conducted by performing the procedures as described in Section 2.3 and Section 2.4 using tap water as a surrogate matrix for TWW. Pre- and post-extraction spiked matrix samples were used as QA/QC samples and were prepared by spiking known amounts of the target analytes (700 and 50 µg/L in the particulate and dissolved phase, respectively) and internal standards (33.3 µg/L in the particulate and dissolved phase) prior to and post-sample preparation (extraction and clean-up). The recovery % for each target analyte at the specific fortification amount was calculated from the response (area of peak) of the analyte in the pre-extraction matrix-matched spiked standard solution divided by the response of the analyte in the post-extraction matrix-matched spiked standard solution and multiplied by 100 (Equation (1)). It is noteworthy that when endogenous concentrations are determined in the matrix, their response is subtracted from the total response measured in the fortified matrices (pre- and post-extraction matrix matched standard solutions).

(1) $$\mathrm{Recovery}\left(\%\right)=\frac{{\mathrm{Area}}_{\mathrm{Pre}-\mathrm{extraction}\;\mathrm{spiked}}}{{\mathrm{Area}}_{\mathrm{Post}-\mathrm{extraction}\;\mathrm{spiked}}}\;\times \;100$$

### 2.8. Performance Characteristics of the HPLC-DAD-FLD and GC-MS Method

For each batch, a calibration curve was run to check the instrumental calibration. The calibration curve used for GC-MS analysis included the concentrations of 0.01, 0.1, 1, 2, 5, 10, 20, 50, 100, and 200 µg/L. The calibration curve used for HPLC-DAD-FLD analysis included the concentrations of 1, 10, 20, 50, 75, 100, 200, 400, 1000, and 1500 µg/L. The resulting correlation coefficients of the calibration curves were calculated after each run and were in the range of 0.98–1.00 (Supplementary Information, Table S6).

The method LOD for each target analyte was defined as the concentration of the target analyte in a fortified matrix (pre-extraction spiked) that was equal to three times the average level (3 × S/N (signal to noise ratio)) of the baseline background in proximity to its respective eluting peak. The method LOQ for each target analyte was defined as the concentration that was ten times the same baseline background (10 × S/N). In addition, all positive found peaks were visually checked and confirmed in the samples. The extraction method reproducibility was assessed by using replicate analyses (*n* = 4) at 100 µg/L (Supplementary Information, Table S7; performed with HPLC-DAD-FLD determination). For GC-MS, the variances in the IS response of the actual samples were used for QA/QC (monitoring method reproducibility during GC-MS analysis; Supplementary Information, Table S8). A methanol solvent blank solution was injected after the analysis of 10 consecutive samples to monitor carry-over effects during HPLF-DAD-FLD and GC-MS analysis.

### 2.9. Calculation of Specific PAH Forensic Ratios

The PAH concentrations measured in the particulate and dissolved phases were combined to obtain the total concentration based on the original weight or volume of the particulate and dissolved phase, respectively. From the determined total concentrations of ANT, PHE, FLT, PYR, BaA, CHR, IND, and BgP in the TWW samples, the forensic ratios were calculated (Supplementary Information, Table S9). The ratios from these specific PAHs were established based on the varying occurrence of PAHs in samples from different emission sources, e.g., type of combustion [12].

### 2.10. Data Analysis

For the integration of the target analyte peaks from the GC-MS analysis, the MSD ChemStation E.01.01.335 was used. The target analyte peaks from HPLC-DAD-FLD were integrated with the program ChemStation software version A.01.08.108. Further data processing was performed in Excel 2021. The internal standards method was used for quantification for both GC-MS and HPLC-DAD-FLD determination (Supplementary Information, Table S10).

## 3. Results and Discussion

### 3.1. Method Performance

A high extraction efficiency was observed in this study for the particulate phase (Table 2). This was attributed to the high temperature and pressure of the ASE procedure, which increases dissolution and analyte desorption from the particle matrix and facilitates the liquid to more efficiently come into contact with the particle’s surface [28]. The absolute recoveries for the PAHs are presented in Table 3. The absolute recoveries ranged from 57% (NAP) to 104% (IND) for the particulate phase, and from 42% (ANT) to 79% (BgP) for the dissolved phase. Particulate PAH recoveries overlap with previously reported recoveries in the literature. In the dissolved phase, PAHs recoveries are somewhat lower than previous reports, but they were acceptable as fit-for-purpose (Table 2). The recoveries reported from the literature in the dissolved phase were compared to those herein (two-sample t-test at significance level of 0.05) considering a 20% RSD (uncertainty, relative standard deviation) for all values.

When comparing the two separation techniques, GC separated the PAH compounds in 34.75 min, while HPLC compounds were separated in 22.50 min. With HPLC determination, there were analytical drawbacks for FLU analysis due to baseline drifts. This is a phenomenon where the baseline increases or decreases in intensity when the wavelength of the FLD switches [35]. Hence, no values are presented for this target analyte in the list of absolute recoveries for the water phase. DBA and IND were only detected in one of the water samples; therefore, absolute recoveries were not calculated for these target analytes, and these analogues were semi-quantified.

The reproducibility of GC-MS and HPLC-DAD-FLD determinations were mainly within the set boundary of 20% RSD (Supplementary Information, Tables S7 and S8). For GC-MS, due to different amounts of IS added to the samples, the RSD% values were calculated for four different IS concentrations (5, 10, 50, and 100 µg/L) and also differentiated by particulate and dissolved samples. F-PHE had an RSD% < 20% in all concentrations, while the RSD% was <20% in four out of five IS concentrations for F-Bisphenyl (except dissolved phase, 50 µg/L IS) and F-CHR (except particulate phase, 5 µg/L IS). In the case of HPLC-DAD-FLD, the RSD% of the 100 µg/L calibration standard stayed below 9.0% for all compounds and ISs, except for ANT (22%) and DBA (>30%). Due to the large deviation of DBA, the target analyte was semi-quantified.

Overall, all 16 target PAHs were determined with GC-MS, while the HPLC-DAD-FLD determined 14 PAHs; BaP and the IS F-BkF were not baseline chromatographically separated with HPLC. The LODs and LOQs of the PAHs are summarized in Supplementary Information, Table S13. The GC-MS showed low LODs for 13 out of 16.

PAHs were observed even for those target analytes that were not detectable by HPLC-DAD-FLD. It is noteworthy that, for IND, where GC-MS demonstrated LODs of 0.0030 µg/g and 0.060 µg/L for particulate and dissolved phase, respectively, HPLC-DAD-FLD demonstrated LODs higher by one order of magnitude (0.030 µg/g and 0.60 µg/L for particulate and dissolved phase, respectively). In the cases of ACE and PYR, both methods maintained LODs of 0.00030 µg/g and 0.0060 µg/L for particulate and dissolved phase, respectively. For BkF, HPLC-DAD-FLD demonstrated the lowest LOD, with 0.00030 µg/g for particulates and 0.0060 µg/L for the dissolved phase. Typical GC-MS and HPLC-DAD-FLD chromatograms from fortified concentrations of 100 µg/L in calibration standards are presented in Figure 4 and Figure 5 and in Supplementary Information, Figure S1.

### 3.2. Application of Methods

#### 3.2.1. Performance

The methodology was applied to TWW samples collected from tunnels in Oslo and Trondheim (*n* = 5). GC-MS determination demonstrated that the target analytes with the highest detection rates were PHE and PYR with 80%, followed by FLU 60% (Supplementary Information, Table S14). In addition, NAP, ANT, and FLT demonstrated detection rates of 40%. In total, 8 out of the 16 PAH target analytes were detected in the TWW samples by GC-MS. HPLC-DAD-FLD determination demonstrated detection rates of 80% for NAP, BkF and BgP, followed by PYR and ACE (60% and 40%, respectively; Supplementary Information, Table S9). In total, 8 out of the 16 PAHs were detected in the TWW samples by HPLC-DAD-FLD. Taken together, the two analytical techniques detected 13 out of 16 PAHs. However, the detected PAHs are different between the two methods. FLU, ANT, FLT, BaA, and CHR are only detected by GC-MS, while ACE, BbF, BkF,BgP, and DBA are only detected by HPLC-DAD-FLD. None of the methods detected ACY, BaP, or IND in the TWW samples. The differences in the detection rates of the compounds were attributed to the LODs of the analytical methods. BaA, ANT, FLT, CHR, and PHE were all detected by GC-MS since this technique demonstrated lower LODs for those than HPLC-DAD-FLD (not detected). One exception was PHE being detected by both analytical techniques in the same sample, but the highest concentration was still detected by GC-MS. This discrepancy was due to PHE elution, where there is a baseline shift during HPLC-FLD determination, contributing to loss of signal. On the contrary, BkF was only detected by HPLC-DAD-FLD, which in this case showed lower LOD than GC-MS. In the case of ACE, both analytical techniques demonstrated the same LODs, but only HPLC-FLD detected the compound (false positive). For BbF, BgP, DBA, and NAP, HPLC-DAD-FLD demonstrated higher LODs than GC-MS, but nonetheless, peaks were detected solely or in higher concentrations with HPLC-DAD-FLD, indicating the occurrence of false positive peaks or/and co-eluting components. While the FLD receives the light emitted from the compounds eluting at the expected elution time, the MS detector measures the compounds’ mass/charge ratio [36]. This gives the MS detector the ability to differentiate between specific chemical structures, which is not always possible with FLD. If endogenous compounds in the sample matrix coelute with BbF, BgP, DBA, and NAP and are fluorescent, the HPLC-DAD-FLD method cannot differentiate amongst those, leading to false positive findings. Co-eluting compounds could for instance be alkylated PAHs or heteroatom polycyclic aromatic compounds, which are co-occurring in tunnel wash water [37]. Eventually, the GC-MS method was the preferred method for the determination of PAHs, while HPLC-DAD-FLD was used for cross-array confirmation of the GC-MS data.

#### 3.2.2. Concentrations in Particulate and Dissolved Phases

The concentrations of the target PAHs were measured in the particle (>0.45 µm) and dissolved (<0.45 µm) phase of the TWW samples (Supplementary Information, Tables S15 and S16). Based on these values, the total PAH concentrations in each TWW sample were calculated by normalizing to the original weight or volume (Table 3).

The particulate phase contained several PAHs. Similar results were observed by Rabodonirina et al. [38] in freshwater systems, where the particulate and sediment phases contained most of the target PAHs, and 4-, 5-, and 6-ringed PAHs constituted the main portion of the total PAH concentrations. From this point onwards, 2- and 3-ringed PAHs are termed LMW PAHs, 4-ringed are termed intermediate molecular weight (IMW) PAHs, and 5- and 6-ringed PAHs are termed HMW PAHs, based on the definition of Abdel-Shafy and Mansour [39]. In the present study, the HMW PAHs BbF, BkF, BgP, and DBA were observed in at least one of the five particulate TWW samples, and the detected concentrations were in the range of 0.33 to 68 µg/g. Among the LMW PAHs (including NAP, ACY, ACE, FLU, PHE, and ANT), only FLU and ANT were detected (≤2.0 µg/g). The IMW PAHs, FLT, PYR, BaA, and CHR, were all detected in at least one sample (range: 0.16 to 6.6 µg/g). Allan et al. [40] and Meland [41] also detected PAHs in the particulate phase of TWW (Supplementary Information, Table S17). The HMW PAHs were detected in the range of 0.043 to 0.93 µg/g. LMW PAHs were detected in the range of 0.020 to 1.0 µg/g, and IMW PAHs were present in the range of 0.075 to 2.0 µg/g. Allan et al. [40] detected NAP, ACY, and ACE in the tunnel wash water, while these were not detected by Meland [41] or in the present study. These are all LMW PAHs and have potentially lower affinity towards the particulate phase since they are considered to be polar compared to heavier PAHs.

PAHs were also detected in the dissolved phase of the TWW samples. In contrast to the particulate phase, the dissolved phase mainly contained LMW PAHs in the range of 0.0098 to 0.50 µg/L. This was attributed to the decreased water solubility (higher octanol-water partitioning coefficient) with increased molecular weight of PAHs [42]. NAP and PHE were both detected in four out of five samples. ACE was found in three out of five samples. ANT was detected in one sample out of the five, while ACY and FLU were not detected in these samples. From the IMW and HMW PAHs, only FLT, PYR and BkF were detected, and the concentrations were in the range of 0.0069 to 0.15 µg/L. TWW contains several other factors that can influence the sorption of PAHs to particulate matter, such as high ion concentrations (e.g., 140 to 220 mg/L Na^+^ and 150 to 260 mg/L Cl^−^) and the presence of surfactants.

Organic and inorganic colloids that are <0.45 µm can be associated with PAHs, pass through the pore filter, and consequently, be detected in the dissolved phase. Paruch and Roseth [43] analyzed the dissolved phase from TWW and detected the LMW PAHs (NAP, ACY, FLU, and PHE) in the range of 0.060 to 1.2 µg/L; BaP and IND (HMW PAHs) in the range of 0.080 to 0.36 µg/L; and IMW FLT and PYR in the range of 0.37 to 0.61 µg/L (Supplementary Information, Table S17). The reported concentrations from the literature indicated the same trend as found in this study, where most of the LMW PAH compounds were present, while the HMW compounds (BkF in this study, and BaP and IND in the study of Paruch and Roseth [43]) had low detection rates in water due to by-default lower solubility. The few HMW PAHs that were detected in the TWW were categorized as acutely toxic to aquatic life upon exposure based on the quality standard classifications from the Norwegian Environmental Agency [44].

#### 3.2.3. Forensic Ratios

The main purpose of the use of the forensic ratios is to describe the different origins of the PAH compounds in the samples [45]. One such ratio is ANT/(ANT + PHE), which differentiates between petrogenic, i.e., low temperature, sources (<0.10) and pyrogenic, i.e., high temperature, sources (>0.10) (Supplementary Information, Table S9). For the TWW samples from Bjørnegård (BT), Smestad (Sme), and Grillstad (Gri), the forensic ratio ANT/(ANT + PHE) was >0.1, indicating the incomplete combustion of crude oil refinery products as the origin of the PAHs. Ratios in samples from Strindheim (Str) and Granfoss (Gra) indicated a petroleum source that was not exposed to high temperatures (Table 4).

The FLT/(FLT + PYR) ratio is another source indicator, separating between non-combusted sources, low temperature processes, and high temperature processes (Supplementary Information, Table S9). For the BT, Sme, and Gri samples, this ratio also indicated that petroleum was the source of origin of PAHs at these sites (<0.33), while high temperature processes such as grass, wood, and coal combustion were a potential PAH source for Gra tunnel. Traffic emissions involve both combusted and non-combusted forms of petroleum products, which was reflected in the ratios. It was not possible to calculate the FLT/(FLT + PYR) ratio for Str, as FLT and PYR were not detected in this sample.

The ratio BaA/(BaA + CHR) separates between non-combusted petroleum and the combustion of organic matter, with an intermediate range where these sources overlap (Supplementary Information, Table S9). The BaA/(BaA + CHR) ratio provided contradicting results compared to the previous two ratios (Table 4). For BT, it indicated that the PAHs in the sample originated from the combustion of wood, coal, diesel, or gasoline, contrary to the non-combusted petroleum source indicated by the ANT/(ANT + PHE) and FLT/(FLT + PYR) ratios. As for Gra, the BaA/(BaA + CHR) ratio indicated that non-combusted petroleum was the source of origin, while the other two ratios indicated combustion of wood, coal, diesel, or gasoline.

The calculated forensic ratios in this study differed from previously reported calculated ratios in TWW. Allan et al. [40] observed, in particulate phase samples, ANT/(ANT + PHE) ratios of <0.10, FLT/(FLT + PYR) ratios of 0.33 to 0.38, and BaA/(BaA + CHR) ratios of 0.33 to 0.35. All of these indicate low temperature processes or non-combusted petroleum as the source of origin in the different matrices road TWW, river water, snow, and sediments from Oslo (Norway). Yunker et al. [12] summarized the FLT/(FLT + PYR) ratios of 0.41 to 0.49, potentially indicating liquid fossil fuel combustion, and BaA/(BaA + CHR) ratios of 0.39 to 0.60, pointing towards the high-temperature combustion of organic material. It is noteworthy that PAHs can also be susceptible to degradation by photolysis or oxidation from air [46], and degradation can alter these forensic ratios.

## 4. Conclusions

An ASE and a SPE procedure were investigated and used to extract 16 PAHs in the particulate and dissolved phases of TWW samples. ASE yielded absolute recoveries ranging between 57% and 104% for the particulate phase, and SPE yielded absolute recoveries ranging between 42% and 79% for dissolved phase. It can be concluded that both extraction methods had sufficient recoveries and were well suited for sample analysis (fit-for-purpose). Furthermore, the separation and detection capability of GC-MS and HPLC-DAD-FLD for the standard EPA 16 PAHs was demonstrated. GC-MS displayed lower LODs compared to HPLC-DAD-FLD for 13 out of the 16 PAHs by a factor of 2 (BbF, BgP) to 100 (2 orders of magnitude for NAP), while FLU and BaP were not detectable by HPLC-DAD-FLD. The highest differences in LODs among the two techniques were observed for the lighter PAHs (reaching one order of magnitude difference). For ACE and PYR, the LODs were similar in both analytical methods (1.0 µg/L), and BkF demonstrated lower LODs when analyzed by HPLC-DAD-FLD (5-fold). Both analytical techniques showed a trend of increasing LODs with increasing molecular weight of the analytes. Extraction by ASE and SPE and analysis by GC-MS and HPLC-DAD-FLD were successfully utilized to investigate the presence and distribution of PAHs between particulate and dissolved phases in TWW samples from different tunnels. FLU, ANT, FLT, PYR, BaA, CHR, and BkF were detected in the particulate phase, while the occurrence profile of the dissolved phase samples included NAP, ACE, PHE, ANT, FLT, PYR, and BkF. The results were further used to calculate forensic ratios of PAHs to pinpoint the potential sources of pollution. Both combusted and non-combusted petrol sources were indicated as potential emission sources of PAHs.

The present HPLC-DAD-FLD method was 12 min shorter than the GC-MS method, and it performed in an optimal manner for the detection of heavier PAH compounds. However, the GC-MS determination was more selective, especially when the matrix contains isobaric compounds that can be co-extracted and eluted at the same time as the target analytes. Therefore, based on the results in this study, we recommend using GC-MS for the detection of PAHs in TWW samples.

## Figures and Tables

**Table 1:** Conditions set for the detection by HPLC-FLD. PMT = photomultiplier tube.

Time Interval (min)	FLD Excitation (nm)	FLD Emission (nm)	PMT
0.00–6.75	230	352	10
6.75–7.50	260	352	10
7.50–15.50	230	420	10
15.50–16.80	290	430	10
16.80–22.50	230	460	10

**Table 2:** Summary of the absolute recoveries (%; mean values) for ASE (for particulate phase extraction) and SPE (for dissolved phase extraction). The recoveries are compared to reports from literature. n.d. = not detected, n.a. = not analyzed.

	Recoveries ASE (%) for Particle Phase			Recoveries SPE (%) for the Dissolved Phase			
	Present Study [29]	Alexandrou et al. [30], *n* = 5	Wang et al. [31], *n* = 3	Present Study	Oleszczuk and Baran [32], *n* = 9	Kootstra et al. [33], *n* = 3	Bruzzoniti et al. [34], *n* = 6
NAP	57	n.a.	70	54	99 ^a^	88 ^a^	n.d.
ACY	n.d.	92	85	60	104 ^a^	82	n.a.
ACE	73	83	91	51	64	81	21 ^a^
FLU	78	89	89	n.a.	93	84	67
PHE	82	94	96	44	72 ^a^	83 ^a^	92 ^a^
ANT	83	85	93	42	82 ^a^	77 ^a^	81 ^a^
FLT	87	90	84	47	81 ^a^	71	85 ^a^
PYR	88	90	97	46	90 ^a^	69	88 ^a^
BaA	91	97	90	59	106 ^a^	68	84
CHR	91	99	77	65	96 ^a^	75	82
BbF	91	102	94	68	91	64	82
BkF	91	109	105	71	87	66	77
BaP	92	96	110	68	77	51	74
DBA	88	105	112	n.d.	72	64	70
BgP	92	107	111	79	69	65	74
IND	104	99	73	n.d.	81	58	70

^a^ The recovery of the literature study is statistically different from the present study (two-sample *t*-test). Results from the two-sample *t*-test are summarized in Supplementary Information, Tables S11 and S12.

**Table 3:** Detected total concentrations (µg/L) of 16 PAH compounds in tunnel wash water samples from 5 different tunnels in Norway (BT = Bjørnegård tunnel; Str = Strindheim tunnel; Sme = Smestad tunnel; Gra = Granfoss tunnel; Gri = Grillstad tunnel). n.d. = not detected, n.a = not analyzed, and * indicates values that are false positives.

PAH	BT		Str		Sme		Gra		Gri	
	GC-MS µg/L	HPLC-FLD µg/L	GC-MS µg/L	HPLC-FLD µg/L	GC-MS µg/L	HPLC-FLD µg/L	GC-MS µg/L	HPLC-FLD µg/L	GC-MS µg/L	HPLC-FLD µg/L
NAP	0.25	0.50	n.d.	0.031	n.d.	0.10	0.074	0.47	n.d.	n.d.
ACY	n.d.	n.d.	n.d.	n.d.	n.d.	n.d.	n.d.	n.d.	n.d.	n.d.
ACE	n.d.	n.d.	n.d.	n.d.	n.d.	0.0098	n.d.	0.21	n.d.	0.012
FLU	n.d.	n.a	1.1	n.a	0.44	n.a	n.d.	n.a	0.58	n.a
PHE	0.091	0.022	n.d.	n.d.	0.026	n.d.	0.032	n.d.	0.034	n.d.
ANT	n.d.	n.d.	0.12	n.d.	n.d.	n.d.	9.3	n.d.	n.d.	n.d.
FLT	0.15	n.d.	n.d.	n.d.	n.d.	n.d.	3.9	n.d.	n.d.	n.d.
PYR	6.3	5.1	n.d.	n.d.	0.088	6.3	1.4	n.d.	1.5	18
BaA	1.84	n.d.	n.d.	n.d.	n.d.	n.d.	n.d.	n.d.	n.d.	n.d.
CHR	n.d.	n.d.	n.d.	n.d.	n.d.	n.d.	6.3	n.d.	n.d.	n.d.
BbF	n.d.	4.3 *	n.d.	n.d.	n.d.	n.d.	n.d.	n.d.	n.d.	n.d.
BkF	n.d.	n.d.	n.d.	0.0069	n.d.	0.038	n.d.	0.0075	n.d.	3.0
BaP	n.d.	n.a	n.d.	n.a	n.d.	n.a	n.d.	n.a	n.d.	n.a
BgP	n.d.	5.2 *	n.d.	n.d.	n.d.	280 *	n.d.	230 *	n.d.	230 *
IND	n.d.	n.d.	n.d.	n.d.	n.d.	n.d.	n.d.	n.d.	n.d.	n.d.
DBA	n.d.	n.d.	n.d.	n.d.	n.d.	n.d.	n.d.	n.d.	n.d.	400 *
Total PAH	0.52	0.52	0.12	0.038	0.11	0.15	0.11	0.69	0.034	0.022

**Table 4:** Calculated forensic ratios based on the total measured concentrations of the 16 EPA PAHs in the five tunnel wash water samples BT, Str, Sme, Gra, and Gri. The ratios are based on the PAHs of ANT, PHE, FLT, PYR, BaA, and CHR. n.d. = not detected.

Sampling Site	ANT/(ANT + PHE)	FLT/(FLT + PYR)	BaA/(BaA + CHR)
BT	0.00	0.02	1.00
Str	1.00	n.d.	n.d.
Sme	0.00	0.00	n.d.
Gra	1.00	0.73	0.00
Gri	0.00	0.00	n.d.

## Data Availability

The data for this study can be found in this article or in the Supplementary Materials.

## Supplementary Materials

The following supporting information can be downloaded at: https://www.mdpi.com/article/10.3390/toxics10070399/s1, Table S1: Information concerning location in Norway, approximate length of the tunnel, average annual daily traffic, and speed limits in the five tunnels where the tunnel wash water samples were collected; Table S2: Values for different run parameters used in the accelerated solvent extraction (ASE) of PAHs in particulate phase samples; Table S3: Applied temperature gradient for GC-MS analysis; Table S4: Settings for the mass spectrometry detector for PAH analysis, Table S5: HPLC-DAD-FLD conditions used during solvent gradient separation of 16 PAHs; Table S6: Calibration curve correlation coefficients for different batch analyses with GC-MS and HPLC-DAD-FLD; Table S7: Extraction method reproducibility data at a fortification amount of 100 μg/L (*n* = 4; performed with HPLD-FLD analysis); Table S8: Relative standard deviations (RSD%) for the internal standards (IS): F-Bisphenyl, F-PHE, and F-CHR analyzed by GC-MS; Table S9: Diagnostic ratios used for PAH compounds to indicate the sources of pollution; Table S10: Summary of which internal standard compound was used to determine each of the target analyte PAHs; Table S11: Summary of average recoveries, relative standard deviations (RSD) and p-value from two-sample t-test for dissolved phase recoveries; Table S12: Summary of average recoveries, relative standard deviations (RSD) and p-value from two-sample t-test for particulate phase recoveries; Table S13: Limits of detection (LOD) and limits of quantification (LOQ) of the 16 PAH compounds. Analyzed with HPLC-FLD and GC-MS; Table S14: Detection rates (DR), median, mean, minimum (Min) and maximum (Max) of the total concentrations (µg/L) for the 16 polycyclic aromatic hydrocarbons (PAH) compounds in the tunnel wash water samples (*n* = 5 analyzed by the two different methods (Gas Chromatography Mass Spectrometry (GC-MS) and High-Performance Liquid Chromatography (HPLC-FLD)); Table S15: Detected concentrations (µg/g) of 16 PAH compounds in the particulate phase of tunnel wash water samples from 5 different tunnels in Norway; Table S16: Detected concentrations (µg/L) of 16 PAH compounds in the dissolved phase of tunnel wash water samples from 5 different tunnels; Table S17: Summary of PAH concentrations detected in tunnel wash water samples in other studies; Figure S1: HPLC-DAD chromatogram presenting the elution order of the target analyte ACY (peak eluting as number three from the left). The sample was a calibration standard sample with a concentration of 100 µg/L of each PAH target analyte.
