# Supplementary material for: The Determination of Polycyclic Aromatic Hydrocarbons (PAHs) with HPLC-DAD-FLD and GC-MS Techniques in the Dissolved and Particulate Phase of Road-Tunnel Wash Water: A Case Study for Cross-Array Comparisons and Applications
Source: Toxics. 2022 Jul 19;10(7):399. doi: 10.3390/toxics10070399 (PMC9321833; doi:10.3390/toxics10070399)
Supplement: Supplementary file 1 [file toxics-10-00399-s001.zip › toxics-1797725-supplementary.pdf]

Supplementary Materials: The Determination of Polycyclic Aromatic Hydrocarbons (PAHs) with HPLC-DAD-FLD and GC-MS Techniques in the Dissolved and Particulate Phase of Road-Tunnel Wash Water: A Case Study for Cross-Array Comparisons and Applications

Hanne Vistnes, Nadine A. Sossalla, Anna Røsvik, Susana V. Gonzalez, Junjie Zhang, Thomas Meyn and Alexandros G. Asimakopoulos

Table S1. Information concerning location in Norway, approximate length of the tunnel, average annual daily traffic, and speed limits in the five tunnels where the tunnel wash water samples were collected. AADT = annual average daily traffic.

Tunnel	Location	Length (m)	AADT (vehicle/day)	Speed limit (km/h)
Bjørnegård (BT)	European Road 16, Bærum	2,200	30,000	80
Granfoss (Gra)	State Highway 150, Olso	2,350	31,000	60
Smestad (Sme)	State Highway 150, Oslo	494	23,000	60
Grillstad (Gri)	European Road 6, Trondheim	748	22,000	80
Strindheim (Str)	State Highway 706, Trondheim	2,597	21,120	60

Table S2. Values for different run parameters used in the accelerated solvent extraction (ASE) of PAHs in particulate phase samples.

System Information	Value
Pressure	1.500 psi
Oven Temperature	100 °C
Cell Size	10 mL
Static Extraction Time	5 min
Number of Static Cycles	2
Rinse Volume	6 mL
Nitrogen Purge	90 sec
Extraction Time	19 min

Table S3. Applied temperature gradient for GC-MS analysis.

Time (min)	Temperature (°C)	Temperature increase rate (°C/min)
0	50	
2	50	
10	250	25
11	250	
23	286	3
26	286	
28.75	308	8
29.75	308	
31.75	310	1
34.75	310	

Table S4. Settings for the mass spectrometry detector for PAH analysis

Time Interval (min)	m/z	PAH Compound
0.00	128	NAP
8.25	172	F-Bisphenyl
9.25	152	ACY
	154	ACE
9.80	166	FLU
10.80	178	PHE, ANT
	196	F-PHE
13.00	202	FLT, PYR
17.00	246	F-CHR
17.50	228	BaA, CHR
19.00	252	BkF, BaP
26.00	276	BgP, IND
	278	DBA

Table S5. HPLC-DAD-FLD conditions used during solvent gradient separation of 16 PAHs.

Time (min)	v/v % Water	v/v % Acetonitrile
0.00	60.0	40.0
0.50	60.0	40.0
8.00	20.0	80.0
11.0	20.0	80.0
13.0	0.00	100
18.0	0.00	100
18.1	60.0	40.0
22.5	60.0	40.0

Table S6. Calibration curve correlation coefficients for different batch analyses with GC-MS and HPLC-DAD-FLD. n.d. = not detected, n.a. = not analyzed.

	GC-MS		HPLC-DAD-FLD		
	Run 1	Run 2	Run 1	Run 2	Run 3
NAP	0.9994	0.998	0.9999	0.9999	0.9999
ACY	0.994	0.9991	0.9999	0.9999	0.9999
ACE	0.993	0.997	0.9993	0.9993	0.9992
FLU	0.9992	0.9998	n.a.	n.a.	n.a.
PHE	0.9999	0.9996	0.9992	0.9998	0.9999
ANT	0.9997	0.9993	0.9999	1.00	0.998
FLT	0.991	0.9990	1.00	1.00	1.00
PYR	0.9999	0.9993	0.9999	0.9999	0.9999
BaA	0.9994	0.9979	0.9999	0.9999	1.00
CHR	0.9994	0.998	0.9999	0.9999	0.9999
BbF	0.996	0.997	0.9999	1.00	0.9999
BkF	0.98	0.9995	0.9998	0.9999	0.9998
BaP	0.997	0.997	n.d.	n.d.	n.d.
IND	0.9997	0.997	0.997	0.993	n.d.
DBA	0.9991	0.998	0.9997	0.9998	0.98
BgP	0.997	0.995	0.9999	1.00	0.9994

Table S7. Extraction method reproducibility data at a fortification amount of 100 μg/L (n = 4; performed with HPLD-FLD analysis). n.d. = not detected.

	RSD%
NAP	0.36
F-NAP	0.58
ACY	0.99
ACE	0.46
PHE	8.9
ANT	22
FLT	0.87
PYR	3.0
F-PYR	1.0
BaA	0.35
CHR	0.38
F-CHR	0.93
BpF	1.7
BkF	0.88
BaP	2.4
DBA	74
BgP	4.8
IND	n.d.

Table S8. Relative standard deviations (RSD%) for the internal standards (IS): F-Bisphenyl, F-PHE, and F-CHR analyzed by GC-MS; DP = dissolved phase, PP = particulate phase.

	F-Bisphenyl	F-PHE	F-CHR
**DF 100 ppb IS** (n=9)	13	10	12
**DF 50 ppb IS** (n=6)	36	9.3	11
**PF 5 ppb** (n=8)	19	19	21
**PF 10 ppb** (n=4)	20	20	20
**PF 50 ppb** (n=13)	15	18	11

Table S9. Diagnostic ratios used for PAH compounds to indicate the sources of pollution. ANT = anthracene, PHE = phenanthrene, FLT = fluoranthene, PYR = pyrene, BaA = benzo(a)anthracene, and CHR = chrysene.

Ratio	Range	Indicated source	Ref.
ANT/(ANT+PHE)	0 – 0.1	Petrogenic/petroleum source	[1]
	0.1 - 1	Pyrogenic/combustion source	[1]
FLT/(FLT+PYR)	0 – 0.33	Fossil fuels (not combusted)	[2]
	0.33 – 0.5	Low temperature process	[2]
	0.5 – 1	High temperature process	[2]
	0 – 0.4	Petroleum (not combusted)	[3]
	0.4 – 0.5	Liquid fossil fuel (car and crude oil) combustion	[3]
	0.5 - 1	Grass, wood, and coal combustion	[3]
BaA/(BaA+CHR)	0 – 0.2	Petroleum (not combusted)	[3]
	0.2 – 0.35	Petroleum (not combusted) or combustion (coal, gasoline)	[3]
	0.35 - 1	Combustion (wood, coal, diesel, gasoline)	[3]
IND/(IND+BgP)	0 – 0.2	Petroleum (not combusted)	[3]
	0.2 – 0.5	Liquid fossil fuel (car and crude oil) combustion	[3]
	0.5 - 1	Grass, wood, and coal combustion	[3]

Table S10. Summary of which internal standard compound was used to determine each of the target analyte PAHs. n.a. = not analyzed.

Compound	GC-MS method	HPLC-DAD-FLD method
NAP	F-Biphenyl	F-NAP
ACY	F-Biphenyl	F-NAP
ACE	F-Biphenyl	F-NAP
FLU	F-PHE	n.a.
PHE	F-PHE	F-NAP
ANT	F-PHE	F-PYR
FLT	F-PHE	F-PYR
PYR	F-PHE	F-PYR
BaA	F-CHR	F-CHR
CHR	F-CHR	F-CHR
BbF	F-CHR	F-CHR
BkF	F-CHR	F-CHR
BaP	F-CHR	F-CHR
DBA	F-CHR	F-CHR
BgP	F-CHR	F-CHR
IND	F-CHR	F-CHR

Table S11. Summary of average recoveries, relative standard deviations (RSD) and p-value from two-sample t-test for dissolved phase recoveries. n.a. = not analyzed, n.d. = not detected.

	**Our study (n=3)**		**Oleszczuk et al. (n=9)**			**Kootstra et al. (n=3)**			Bruzzoniti et al. (n=6)		
	**Average**	**RSD**	**Average**	**RSD**	**p-val.**	**Average**	RSD	p-val.	Average	RSD	p-val.
NAP	54	20	99	20	0.0043	88	20	0.046	n.d.	-	-
ACY	60	20	104	20	0.0067	82	20	0.134	n.a.	-	
ACE	51	20	64	20	0.14	81	20	0.053	21	20	0.00033
FLU	n.d.	n.d.	93	20	-	84	20	-	67	20	-
PHE	44	20	72	20	0.011	83	20	0.023	92	20	0.0041
ANT	42	20	82	20	0.0027	77	20	0.026	81	20	0.0065
FLT	47	20	81	20	0.0070	71	20	0.071	85	20	0.010
PYR	46	20	90	20	0.0026	69	20	0.074	88	20	0.0068
BaA	59	20	106	20	0.0050	68	20	0.44	84	20	0.053
CHR	65	20	96	20	0.028	75	20	0.43	82	20	0.16
BbF	68	20	91	20	0.075	64	20	0.73	82	20	0.25
BkF	71	20	87	20	0.18	66	20	0.68	77	20	0.59
BaP	68	20	77	20	0.39	51	20	0.16	74	20	0.58
DBA	n.d.	n.d.	72	20	-	64	20	-	70	20	-
BgP	79	20	69	20	0.32	65	20	0.30	74	20	0.65
IND	n.d.	n.d.	81	20	-	58	20	-	70	20	-

Table S12. Summary of average recoveries, relative standard deviations (RSD) and p-value from two-sample t-test for particulate phase recoveries. n.a. = not analyzed, n.d. = not detected.

	Our study (n=3)		Alexandrou et al. (n=5)			Wang et al. (n=3)		
		RSD	Average	RSD	p-val.	Average	RSD	p-val.
NAP	57	20	n.a.	-	-	70	20	0.28
ACY	n.a.	n.a.	92	20	-	85	20	-
ACE	73	20	83	20	0.42	91	20	0.25
FLU	78	20	89	20	0.41	89	20	0.47
PHE	82	20	94	20	0.4	96	20	0.39
ANT	83	20	85	20	0.88	93	20	0.53
FLT	87	20	90	20	0.83	84	20	0.84
PYR	88	20	90	20	0.88	97	20	0.58
BaA	91	20	97	20	0.68	90	20	0.95
CHR	91	20	99	20	0.59	77	20	0.37
BbF	91	20	102	20	0.47	94	20	0.85
BkF	91	20	109	20	0.28	105	20	0.43
BaP	92	20	96	20	0.78	110	20	0.34
DBA	88	20	105	20	0.29	112	20	0.22
BgP	92	20	107	20	0.35	111	20	0.32
IND	104	20	99	20	0.75	73	20	0.10

Table S13. Limits of detection (LOD) and limits of quantification (LOQ) of the 16 PAH compounds. Analyzed with HPLC-FLD and GC-MS. No LOD or LOQ for benzo(a)pyrene (BaP) are included in the table for HPLC-FLD, since BaP and the internal standard 9-fluorobenzo(k)fluoranthene (F-BkF) overlapped in the retention time. For fluorene (FLU), it was not possible to find the LODs and LOQs for this compound in the HPLC-FLD analysis, due to a jump in the baseline noise. n.d. = not detected, n.a. = not analyzed.

	GC-MS		HPLC-FLD	
	LOD (µg/L)	LOQ (µg/L)	LOD (µg/L)	LOQ (µg/L)
NAP	0.0030	0.010	0.30	1.0
ACY	0.30	1.0	3.0	10
ACE	0.30	1.0	0.30	1.0
FLU	0.30	1.0	n.a.	n.a.
PHE	0.030	0.10	0.30	1.0
ANT	0.30	1.0	3.0	10
FLT	0.30	1.0	3.0	10
PYR	0.30	1.0	0.30	1.0
BaA	0.60	2.0	3.0	10
CHR	0.60	2.0	6.0	20
BbF	1.5	5.0	3.0	10
BkF	1.5	5.0	0.30	1.0
BaP	1.5	5.0	n.d.	n.d.
DBA	3.0	10	15	50
BgP	1.5	5.0	3.0	10
IND	3.0	10	30	100

Table S14. Detection rates (DR), median, mean, minimum (Min) and maximum (Max) of the total concentrations (μg/L) for the 16 polycyclic aromatic hydrocarbons (PAH) compounds in the tunnel wash water samples (n = 5 analyzed by the two different methods (Gas Chromatography Mass Spectrometry (GC-MS) and High-Performance Liquid Chromatography (HPLC-FLD)). n.d. = not detected.

	DR (%)		Median (μg/L)		Mean (μg/L)		Min (μg/L)		Max (μg/L)	
	GC-MS	HPLC-FLD	GC-MS	HPLC-FLD	GC-MS	HPLC-FLD	GC-MS	HPLC-FLD	GC-MS	HPLC-FLD
NAP	40	80	n.d.	0.10	0.066	0.22	n.d.	n.d.	0.25	0.50
ACY	n.d.	n.d.	n.d.	n.d.	n.d.	n.d.	n.d.	n.d.	n.d.	n.d.
ACE	n.d.	40	n.d.	0.010	n.d.	0.047	n.d.	n.d.	n.d.	0.21
FLU	60	n.d.	0.44	n.d.	0.42	n.d.	n.d.	n.d.	1.1	n.d.
PHE	80	20	0.032	n.d.	0.037	n.d.	n.d.	n.d.	0.091	0.02
ANT	40	n.d.	n.d.	n.d.	1.9	n.d.	n.d.	n.d.	9.3	n.d.
FLT	40	n.d.	n.d.	n.d.	0.81	n.d.	n.d.	n.d.	3.9	n.d.
PYR	80	60	1.4	5.1	1.9	5.9	n.d.	n.d.	6.3	18
BaA	20	n.d.	n.d.	n.d.	0.37	n.d.	n.d.	n.d.	1.8	n.d.
CHR	20	n.d.	n.d.	n.d.	n.d.	n.d.	n.d.	n.d.	6.2	n.d.
BbF	n.d.	20	n.d.	n.d.	n.d.	0.86	n.d.	n.d.	n.d.	4.3
BkF	n.d.	80	n.d.	0.0075	n.d.	0.62	n.d.	n.d.	n.d.	3.0
BaP	n.d.	n.d.	n.d.	n.d.	n.d.	n.d.	n.d.	n.d.	n.d.	n.d.
BgP	n.d.	80	n.d.	225	n.d.	148	n.d.	n.d.	n.d.	277
IND	n.d.	n.d.	n.d.	n.d.	n.d.	n.d.	n.d.	n.d.	n.d.	n.d.
DBA	n.d.	20	n.d.	n.d.	n.d.	81	n.d.	n.d.	n.d.	404

Table S15. Detected concentrations (μg/g) of 16 PAH compounds in the particulate phase of tunnel wash water samples from 5 different tunnels in Norway (BT = Bjørnegård tunnel, Str =Strindheim tunnel, Sme = Smestad tunnel, Gra = Granfoss tunnel, Gri = Grillstad tunnel). n.a. = not analyzed, n.d. = not detected, and * indicates values that are false positives.

PAH	BT		Str		Sme		Gra		Gri	
	GC-MS μg/g	HPLC-FLD μg/g	GC-MS μg/g	HPLC-FLD μg/g	GC-MS μg/g	HPLC-FLD μg/g	GC-MS μg/g	HPLC-FLD μg/g	GC-MS μg/g	HPLC-FLD μg/g
NAP	n.d.	n.d.	n.d.	n.d.	n.d.	n.d.	n.d.	n.d.	n.d.	n.d.
ACY	n.d.	n.d.	n.d.	n.d.	n.d.	n.d.	n.d.	n.d.	n.d.	n.d.
ACE	n.d.	n.d.	n.d.	n.d.	n.d.	n.d.	n.d.	n.d.	n.d.	n.d.
FLU	n.d.	n.a.	1.2	n.a.	0.11	n.a.	n.d.	n.a.	0.063	n.a.
PHE	n.d.	n.d.	n.d.	n.d.	n.d.	n.d.	n.d.	n.d.	n.d.	n.d.
ANT	n.d.	n.d.	n.d.	n.d.	n.d.	n.d.	2.0	n.d.	n.d.	n.d.
FLT	n.d.	n.d.	n.d.	n.d.	n.d.	n.d.	0.84	n.d.	n.d.	n.d.
PYR	6.6	5.3	n.d.	n.d.	n.d.	1.5	0.30	n.d.	0.16	2.0
BaA	1.9	n.d.	n.d.	n.d.	n.d.	n.d.	n.d.	n.d.	n.d.	n.d.
CHR	n.d.	n.d.	n.d.	n.d.	n.d.	n.d.	1.3	n.d.	n.d.	n.d.
BbF	n.d.	4.5*	n.d.	n.d.	n.d.	n.d.	n.d.	n.d.	n.d.	n.d.
BkF	n.d.	n.d.	n.d.	n.d.	n.d.	n.d.	n.d.	n.d.	n.d.	0.33
BaP	n.d.	n.a.	n.d.	n.a.	n.d.	n.a.	n.d.	n.a.	n.d.	n.a.
BgP	n.d.	5.4*	n.d.	n.d.	n.d.	68*	n.d.	50*	n.d.	24*
IND	n.d.	n.d.	n.d.	n.d.	n.d.	n.d.	n.d.	n.d.	n.d.	n.d.
DBA	n.d.	n.d.	n.d.	n.d.	n.d.	n.d.	n.d.	n.d.	n.d.	44*
Total PAH	8.5	15	1.2	0.00	0.11	70	4.5	50	0.23	70

Table S16. Detected concentrations (μg/L) of 16 PAH compounds in the dissolved phase of tunnel wash water samples from 5 different tunnels in Norway (BT = Bjørnegård tunnel, Str =Strindheim tunnel, Sme = Smestad tunnel, Gra = Granfoss tunnel, Gri = Grillstad tunnel). n.a. = not analyzed, n.d. = not detected.

PAH	BT		Str		Sme		Gra		Gri	
	GC-MS μg/L	HPLC-FLD μg/L	GC-MS μg/L	HPLC-FLD μg/L	GC-MS μg/L	HPLC-FLD μg/L	GC-MS μg/L	HPLC-FLD μg/L	GC-MS μg/L	HPLC-FLD μg/L
NAP	0.25	0.50	n.d.	0.031	n.d.	0.10	0.074	0.47	n.d.	n.d.
ACY	n.d.	n.d.	n.d.	n.d.	n.d.	n.d.	n.d.	n.d.	n.d.	n.d.
ACE	n.d.	n.d.	n.d.	n.d.	n.d.	0.0098	n.d.	0.21	n.d.	0.012
FLU	n.d.	n.a.	n.d.	n.a.	n.d.	n.a.	n.d.	n.a.	n.d.	n.a.
PHE	0.091	0.022	n.d.	n.d.	0.026	n.d.	0.032	n.d.	0.034	n.d.
ANT	n.d.	n.d.	0.12	n.d.	n.d.	n.d.	n.d.	n.d.	n.d.	n.d.
FLT	0.15	n.d.	n.d.	n.d.	n.d.	n.d.	0.0068	n.d.	n.d.	n.d.
PYR	0.030	n.d.	n.d.	n.d.	0.088	n.d.	n.d.	n.d.	n.d.	n.d.
BaA	n.d.	n.d.	n.d.	n.d.	n.d.	n.d.	n.d.	n.d.	n.d.	n.d.
CHR	n.d.	n.d.	n.d.	n.d.	n.d.	n.d.	n.d.	n.d.	n.d.	n.d.
BbF	n.d.	n.d.	n.d.	n.d.	n.d.	n.d.	n.d.	n.d.	n.d.	n.d.
BkF	n.d.	n.d.	n.d.	0.0069	n.d.	0.038	n.d.	0.0075	n.d.	0.0097
BaP	n.d.	n.a.	n.d.	n.a.	n.d.	n.a.	n.d.	n.a.	n.d.	n.a.
BgP	n.d.	n.d.	n.d.	n.d.	n.d.	n.d.	n.d.	n.d.	n.d.	n.d.
IND	n.d.	n.d.	n.d.	n.d.	n.d.	n.d.	n.d.	n.d.	n.d.	n.d.
DBA	n.d.	n.d.	n.d.	n.d.	n.d.	n.d.	n.d.	n.d.	n.d.	n.d.
Total PAH	0.52	0.52	0.12	0.038	0.11	0.15	0.11	0.69	0.034	0.022

Table S17. Summary of PAH concentrations detected in tunnel wash water samples in other studies. PF = particle fraction suspended in water, SF= sediment fraction, DF = dissolved fraction. n.d. = not detected, n.a. = not analyzed.

	**Nordby [4]**	**Granfoss [4]**	**Vassum [5]**	**Hanekleiv [6]**
Fraction	PF (μg/g)	PF (μg/g)	SF (μg/g)	DF (μg/L)
NAP	0.14	0.099	n.d.	1.15
ACY	0.057	0.048	n.d.	0.06
ACE	0.021	0.02	n.d.	n.a.
FLU	0.074	0.068	0.216	0.07
PHE	1.0	0.76	0.602	0.17
ANT	0.096	0.076	0.119	n.a.
FLT	1.1	0.95	0.386	0.37
PYR	2.0	1.9	0.648	0.61
BaA	0.14	0.11	0.075	n.a.
CHR	0.28	0.2	0.374	n.a.
BbF	0.4	0.3	0.153	n.a.
BkF	0.057	0.043	0.075	n.a.
BaP	0.15	0.16	0.081	0.36
BgP	0.93	0.66	0.237	n.a.
IND	0.17	0.13	0.08	0.08
DBA	0.074	0.064	n.d.	n.a.
Total PAH	6.689	5.588	3.05	2.86

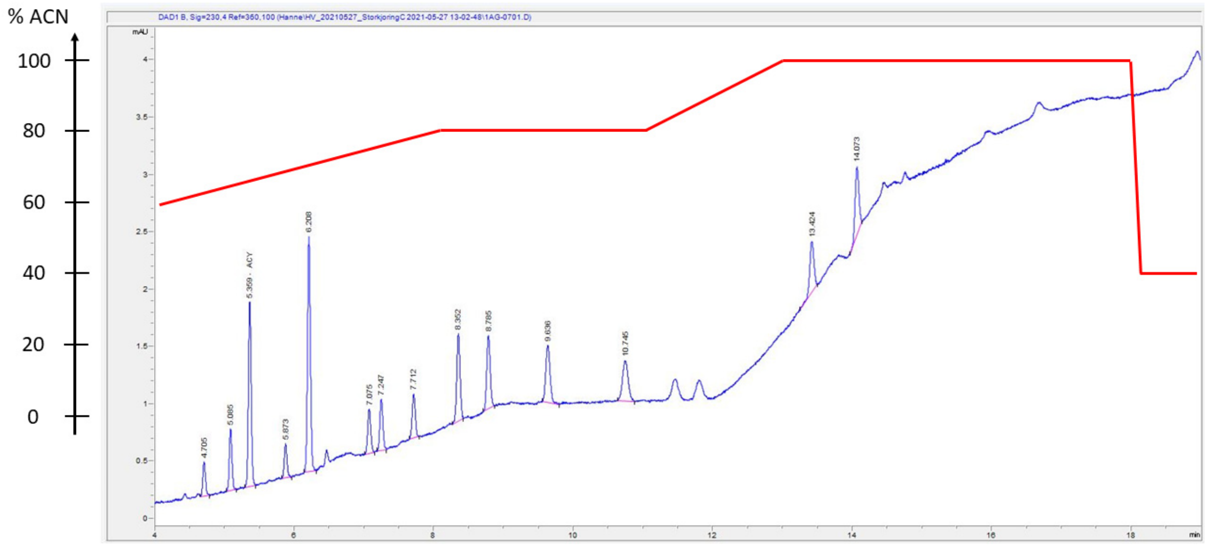

Figure S1. HPLC-DAD chromatogram presenting the elution order of the target analyte ACY (peak eluting as number three from the left). The sample was a calibration standard sample with a concentration of 100 µg/L of each PAH target analyte.

References:

1. Budzinski, H.; Jones, I.; Bellocq, J.; Piérard, C.; Garrigues, P. Evaluation of Sediment Contamination by Polycyclic Aromatic Hydrocarbons in the Gironde Estuary. *Mar. Chem.* **1997**, *58*, 85–97. https://doi.org/10.1016/S0304-4203(97)00028-5.
2. Emsbo-Mattingly, S.; Litman, E. Chapter 34—Forensic Identification of Historical and Ongoing Tar Oil Releases in Nearshore Environments. In *Oil Spill Environmental Forensics Case Studies*; Stout, S.A., Wang, Z., Eds.; Butterworth-Heinemann: 2018, Rockland, MA, USA; pp. 785–826. https://doi.org/10.1016/B978-0-12-804434-6.00034-3.
3. Yunker, M.B.; Macdonald, R.W.; Vingarzan, R.; Mitchell, R.H.; Goyette, D.; Sylvestre, S. PAHs in the Fraser River Basin: A Critical Appraisal of PAH Ratios as Indicators of PAH Source and Composition. *Org. Geochem.* **2002**, *33*, 489–515. https://doi.org/10.1016/S0146-6380(02)00002-5.
4. Allan, I.J.; O'Connell, S.G.; Meland, S.; Bæk, K.; Grung, M.; Anderson, K.A.; Ranneklev, S.B. PAH Accessibility in Particulate Matter from Road-Impacted Environments. *Environ. Sci. Technol.* **2016**, *50*, 7964–7972. https://doi.org/10.1021/acs.est.6b00504.
5. Meland, S. *Chemical Characterisation of Sediment from Vassum Sedimentation Basin*; NPRA Reports; 94; Norwegian Public Roads Administration, 2012, Oslo, Norway; pp. 1–22.
6. Paruch, A.M.; Roseth, R. Treatment of Tunnel Wash Water—Experiments with Organic Sorbent Materials. Part II: Removal of Toxic Metals. *J. Environ. Sci.* **2008**, *20*, 1042–1045. https://doi.org/10.1016/S1001-0742(08)62147-6.
